# A fast parallel algorithm for finding the longest common sequence of multiple biosequences

**DOI:** 10.1186/1471-2105-7-S4-S4

**Published:** 2006-12-12

**Authors:** Yixin Chen, Andrew Wan, Wei Liu

**Affiliations:** 1 Department of Computer Science and Engineering, Washington University in St. Louis, St. Louis, MO 63130, USA; 2 Department of Computer Science, Yangzhou University, Yangzhou 225009, China

## Abstract

**Background:**

Searching for the longest common sequence (LCS) of multiple biosequences is one of the most fundamental tasks in bioinformatics. In this paper, we present a parallel algorithm named FAST_LCS to speedup the computation for finding LCS.

**Results:**

A fast parallel algorithm for LCS is presented. The algorithm first constructs a novel successor table to obtain all the identical pairs and their levels. It then obtains the LCS by tracing back from the identical character pairs at the last level. Effective pruning techniques are developed to significantly reduce the computational complexity. Experimental results on gene sequences in the *tigr *database show that our algorithm is optimal and much more efficient than other leading LCS algorithms.

**Conclusion:**

We have developed one of the fastest parallel LCS algorithms on an MPP parallel computing model. For two sequences *X *and *Y *with lengths *n *and *m*, respectively, the memory required is max{4*(*n*+1)+4*(*m*+1), *L*}, where *L *is the number of identical character pairs. The time complexity is O(*L*) for sequential execution, and *O*(|LCS(*X*, *Y*)|) for parallel execution, where |LCS(*X*, *Y*)| is the length of the LCS of *X *and *Y*. For *n *sequences *X*_1_, *X*_2_, ..., *X*_*n*_, the time complexity is O(*L*) for sequential execution, and *O*(|LCS(*X*_1_, *X*_2_, ..., *X*_*n*_)|) for parallel execution. Experimental results support our analysis by showing significant improvement of the proposed method over other leading LCS algorithms.

## Background

Biological sequence [1] can be represented as a sequence of symbols. For instance, a protein [2] is a sequence of 20 different letters (amino acids), and DNA sequences (genes) can be represented as sequences of four letters A, C, G and T corresponding to the four sub-molecules forming DNA. When a new biosequence is found, we want to know what other sequences it is most similar to. Sequence comparison [3-5] has been used successfully to establish the link between cancer-causing genes and a gene evolved in normal growth and development. One way of detecting the similarity of two or more sequences is to find their LCS.

The LCS problem is to find a substring that is common to two or more given strings and is the longest one of such strings. Since the LCS problem is essentially a special case of the global sequence alignment, all the algorithms for the sequence alignment can be used to solve the LCS problem. Presented in 1981, the Smith-Waterman algorithm [6] is a well known LCS algorithm which was evolved from the Needleman-Wunsch [7] algorithm, and can guarantee the correctness. Aho and et al. [8] gave a lower bound of *O*(*mn*) on time for the LCS problem using a decision tree model. It is shown in [9] that the problem can be solved in *O*(*mn*) time using *O*(*mn*) space by dynamic programming. Mayers and Miller [10] used the technique proposed by Hirschberg [11] to reduce the space complexity to *O*(*m*+*n*) on the premise of the same time complexity.

To further reduce the computation time, some parallel algorithms [12-14] have been proposed for different computational models. For the CREW-PRAM model, Aggarwal [15] and Apostolico et al [16] independently proposed an *O*(log *m *log *n*) time algorithm using *mn*/log *m *processors. Lu et al [17] designed two parallel LCS algorithms, one uses *mn*/log *m *processors with a time complexity of *O*(log^2 ^*n*+log *m*), and the other uses *mn/*(log^2 ^*m *loglog *m*) processors with a running time of *O*(log^2 ^*m *loglog *m*). For the CRCW-PRAM model, Apostolico et al [16] gave an *O*(log *n *(loglog *m*)^2^) time algorithm using *mn*/loglogm processors. Babu and Saxena [18] improved these algorithms for the CRCW-PRAM model. They designed an *O*(log *m*) algorithm with *mn *processors and an O(log^2^*n*) time parallel algorithm. Many parallel LCS algorithms have also been proposed using systolic arrays. Robert et al [19] proposed a parallel algorithm with *n *+ 5*m *steps using *m*(*m*+1) processing elements. Chang et al [20] put forward an algorithm with 4*n*+2*m *steps using *mn *processing elements. Luce et al [21] designed a systolic array with *m*(*m*+1)/2 processing elements and *n*+3*m*+*q *steps where *q *is the length of the LCS. Freschi and Bogliolo [22] addressed the problem of computing the LCS between run-length-encoded (RLE) strings. Their algorithm requires *O*(*m*+*n*) steps on a systolic array of *M*+*N *processing elements, where *M *and *N *are the lengths of the original strings and *m *and *n *are the number of runs in their RLE representation.

For the LCS problem of multiple sequences, the time complexity tends to grow very fast when the number of the sequences increases. For instance, using the Smith-Waterman algorithm to solve the LCS for multiple sequences, the time complexity is , where *n *is the number of sequences, and *n*_*i *_is the length of the *i*th sequence. It is not practicable when *n *is large. Some improvements have been made on the algorithm. The MSA program [23] can process up to ten closely related sequences. It is an implementation of the Carrillo and Lipman algorithm [24] that identifies in advance the portions of the hyperspace not contributing to the solution and excludes them from the computation. Stoye described a new divide and conquer algorithm DCA [25] that extends the capability of MSA. Recently, OMA [26], an iterative implementation of DCA is proposed to speed up the DCA strategy and reduce memory requirements. Based on Feng and Doolittle's algorithm [28], Clustal-W [27] is one of the most widely used multiple sequence alignment software that can also be used for LCS computation.

## Results

In this paper, we present a fast algorithm named FAST_LCS for efficient computation of LCS. The algorithm first seeks the successors of the initial identical character pairs according to a successor table to obtain all the identical pairs and their levels. Then by tracing back from the identical character pair in the last level, it obtains the result of LCS.

The key technique of our algorithm is the use of several effective **pruning operations**. In the process of generating the successors, pruning techniques can remove the identical pairs which can not generate the LCS so as to reduce the search space and accelerate the search speed. The algorithm can be extended to find the LCS of multiple biosequences.

Experimental results on the gene sequences of the *tigr *database, using an MPP parallel computer Shenteng 1800, show that our algorithm can obtain the exact optimal results and is much faster than some other leading LCS algorithms.

## Conclusion

In this paper, we have developed FAST_LCS, one of the fastest parallel LCS algorithms on an MPP parallel computing model. For two sequences *X *and *Y *with lengths *n *and *m*, respectively, the memory complexity of FAST_LCS is max{4*(*n*+1)+4*(*m*+1), *L*}, where *L *is the number of identical character pairs. The time complexity is O*(L*) for sequential execution of the algorithm, and *O*(|LCS(*X*,*Y*)|) for parallel execution, where |LCS(*X*,*Y*)| is the length of the LCS of *X *and *Y*. The algorithm can be extended to solve the LCS for multiple biosequences. For *n *sequences *X*_1_, *X*_2_, ..., *X*_*n*_, the time complexity is O*(L*) for sequential execution, and *O*(|LCS(*X*_1_, *X*_2_, ..., *X*_*n*_)|), which is independent of the number of sequences, for parallel execution. Experimental results support our analysis by showing significant improvement of the proposed method over some other leading LCS algorithms.

## Methods

### The identical character pair and its successor table

Let *X *= (*x*_1_, *x*_2_, ..., *x*_*n*_), *Y *= (*y*_1_, *y*_2_, ..., *y*_*m*_) be two biosequences, where *x*_*i*_, *y*_*i *_∈ {*A*, *C*, *G*, *T*}. We can define an array CH of the four characters so that CH(1) = "*A*", CH(2)= "C", CH(3)= "G" and CH(4)= "T". To find the LCS of *X *and *Y*, we first build the successor tables of the identical characters for the two strings. The successor tables of the identical characters of *X *and *Y *are denoted as *TX *and *TY*, which are two dimensional arrays of size 4(n+1) × 4*(m+1). For sequence *X *= (*x*_1_, *x*_2_, ..., *x*_*n*_), *TX *(*i*, *j*) in table *TX *is defined as follows.



Here, *SX *(*i*, *j*) = {*k*| *x*_*k *_= CH(*i*), *k *>*j*}, where *i *= 1,2,3,4 and *j *= 0,1,...*n*. It can be seen from the definition that if *TX*(*i*, *j*) is not "-", it indicates the position of the next character identical to CH(*i*) after the *j*th position in sequence *X*, otherwise it means there is no such character after the *j*th position.

Example 1

Let *X *= "T G C A T A" and *Y *= "A T C T G A T". Their successor tables *TX *and *TY *are shown in Table 1.

For the sequences *X *and *Y*, if *x*_*i *_= *y*_*j *_= CH(*k*), we call them an identical pair of CH(*k*) and denote it as (*i*, *j*). The set of all the identical character pairs of *X *and *Y *is denoted as *S*(*X*, *Y*).

Let (*i*, *j*) and (*k*, *l*) be two identical character pairs of *X *and *Y*. If *i *<*k *and *j *<*l*, we call (*i*,*j*) a predecessor of (*k*, *l*), or (*k*, *l*) a successor of (*i*, *j*), and denote the relationship as (*i*, *j*) < (*k*, *l*).

Let *P*(*i*, *j*) = {(*r*, *s*) | (*i*, *j*) < (*r*, *s*), (*r*, *s*)∈*S*(*X,Y*)} be the set of all the successors of identical pair (*i*, *j*), if (*k*, *l*)∈*P*(*i*, *j*) and there is no (*k*', *l*')∈*P*(*i*, *j*) satisfying the condition: (*k*', *l*') < (*k*, *l*) and *x*_*k' *_= *x*_*k*_, we call (*k*, *l*) the direct successor of (*i*, *j*), and denote the relationship as (*i*, *j*)≺(*k*, *l*).

If an identical pair (*i*, *j*) ∈ *S*(*X, Y*) and there is no (*k*, *l*)∈*S*(*X, Y*) satisfying (*k*, *l*) < (*i*, *j*), we call (*i*, *j*) an initial identical pair.

For an identical pair (*i*, *j*)∈*S *(*X, Y*), its level is defined as follows:



From the definitions above, the following theorems can be easily deduced:

**Theorem1. **Denote the length of the LCS of *X *and *Y *as |LCS(*X*, *Y*)|, then |LCS(*X*, *Y*)| = max{level (*i*, *j*)|(*i*, *j*)∈*S*(*X*, *Y*)}.

**Proof: **Suppose the identical character pairs corresponding to the longest common subsequence of *X*, *Y *are (*x*_*i*1_, *y*_*j*1_), (*x*_*i*2_, *y*_*j*2_), ..., (*x*_*ir*_, *y*_*jr*_), here *r *= |LCS(*X*, *Y*)|. Since (*i*_1_, *j*_1_) is an initial identical character pair, we have: (*i*_*k*_, *j*_*k*_)≺(*i*_*k*+1_, *j*_*k*+1_), for *k *= 1,2,...,*r*-1, and the level of (*x*_*ik*_, *y*_*jk*_) is *k*. Then we can conclude that the maximal level of all the identical character pairs is *r*, i.e. *r *= max{level(*i*, *j*)|(*i*, *j*)∈*S*(*X*, *Y*)}. The reason is as follows: if *r *is not the maximal level of the identical character pairs of *X*, *Y*, there must be an integer *r*' >*r *and identical character pairs: (*x*_*i*1'_, *y*_*j*1_,)≺(*x*_*i*2'_, *y*_*j*2_,)≺ .....≺(*x*_*ir*_', *y*_*jr*_'). It corresponds to another common subsequence of *X *and *Y *with length *r*' >*r*. This is in contradiction with the condition *r *= |LCS(*X*, *Y*)|.

### The operation of producing successors

In our algorithm, all direct successors of all the initial identical character pairs are first produced in parallel using the successor tables. Then the direct successors of all those successors produced in the first step are generated in parallel. Repeat these operations of generating the direct successors until no more successors could be produced. Therefore, producing all the direct successors for the identical character pairs is a basic operation in our algorithm.

For an identical character pair (*i*, *j*)∈*S*(*X*, *Y*), the operation of producing all its direct successors is as follows:

(*i*,*j*) →{(*TX*(*k,i*),*TY*(*k,j*))|*k *= 1,2,3,4, *TX*(*k,i*)≠'-' and *TY*(*k,j*)≠'-'}     (3)

From (3) we can see that this operation is to couple the elements of the *i*th column of *TX *and the *j*th column of *TY *to get the pairs.

For instance, the successors of the identical character pair (2,5) in Example 1 can be obtained by coupling the elements of the 2^nd ^column of *TX *and the 5^th ^column of *TY*. They are (4, 6), (3, -), (-, -) and (5, 7). Here (3, -) and (-,-) do not represent identical character pairs, they only indicate the end of the process of searching for the successors in the branches they located. After discarding (3,-) and (-,-), the successors of (2, 5) are just (4, 6) and (5, 7).

**Theorem 2. **For an identical character pair (*i*, *j*), the method in (3) above can produce all its direct successors.

**Proof: **By (3), we can produce all direct successors (*TX*(*k*, *i*),*TY*(*k*, *j*)), where *k *= 1,2,3,4, of (*i*, *j*). According to (1),*TX*(*k*, *i*) is the location of the nearest character identical to CH(*k*) after *x*_*i *_in string *X*, and*TY*(*k*, *j*) is the location of the nearest such character of CH(*k*) after *y*_*j *_in string *Y*. This means that identical pairs (*TX*(*k*, *i*),*TY*(*k*, *j*)), where *k *= 1,2,3,4, contain all the direct successors of (*i*, *j*). Consequently, by the same operation on the newly generated identical pairs (*TX*(*k*, *i*),*TY*(*k*, *j*)), where *k *= 1,2,3,4, we can get all of their direct successors. It can be seen that by repeating this operation of producing successors, we can obtain all the successors of (*i*, *j*).

It is obvious that (*TX*(*k*, 0),*TY*(*k*, 0)), where *k *= 1,2,3,4, are all the initial identical pairs of *X *and *Y*. By Theorem 2, we know that starting from these initial identical pairs, all the other identical pairs of *X, Y*, and their levels can be produced.

### The operations of pruning

In the process of generating the successors, pruning techniques can be implemented to remove the identical pairs which can not generate the LCS so as to reduce the search space and improve the efficiency.

#### Pruning Operation 1

If on the same level, there are two identical character pairs (*i*, *j*) and *(k, l) *satisfying (*k*, *l*)*>*(*i*, *j*), then (*k*, *l*) can be pruned without affecting the correctness of the algorithm in obtaining the LCS of *X *and *Y*.

##### Rationale

The reason we can prune (*k*, *l*) is as follows. Suppose the two identical character pairs (*i*, *j*) and (*k*, *l*) are produced by the identical pairs (*i*_1_, *j*_1_) and (*k*_1_, *l*_1_) on the previous level. Let the LCS produced via (*k*_1_, *l*_1_) and (*k*, *l*) be *a*_1_*a*_2_...*a*_*m*_*a*_*m*+1_...*a*_*r*_, here *a*_*m *_corresponds to (*k*_1_, *l*_1_) and *a*_*m*+1 _corresponds to (*k*, *l*). Similarly, let the subsequence produced via (*i*_1_, *j*_1_) and (*i*, *j*) be *b*_1_*b*_2_...*b*_*m*_*b*_*m*+1_...*b*_*s*_...*b*_*q*_, here, *b*_*m *_corresponds to (*i*_1_,*j*_1_) and *b*_*m*+1 _corresponds to (*i*, *j*). Since (*k*, *l*)>(*i*, *j*), by Theorem 2 (*k*, *l*) must be produced after (*i*, *j*). Then there must exist *b*_*s *_(*m*+1 <*s *<*q*) corresponding to (*k*, *l*). Since *a*_*m*+1_...*a*_*r *_and *b*_*s*_*b*_*s*+1_...*b*_*q *_are both the local longest common subsequences obtained by the operations of producing successors on (*k*, *l*), we have "*a*_*m*+1_...*a*_*r*_" = "*b*_*s*_*b*_*s*+1_...*b*_*q*_" which means *q-s = r-(m+1)*, and *q = r*+s-(m+1). Since *s>m+1*, we have *q > r*. Therefore the subsequence "*a*_*m*_*a*_*m*+1_...*a*_*r*_", which is produced on the *m*th level via (*k*_1_, *l*_1_), can not be included in the LCS of *X *and *Y*, and (*k*, *l*) can be pruned without affecting the algorithm to get the LCS of *X *and *Y*.

This pruning operation can be implemented to remove all those redundant identical pairs. After each level of identical pairs are generated, the algorithm checks all the newly generated identical pairs on the same level to find all such identical pairs (*i*, *j*) and (*k*, *l*) satisfying (*k*, *l*) > (*i*, *j*) and then prune (*k*, *l*).

For instance, (4, 6) and (5, 7) in Example 1 are the successors of the identical pair (2, 5). Since they are on the same level and (4, 6)≺(5, 7), we can prune (5, 7).

For another identical character pair (1,2) in Example 1, its successors are (4,6), (3,3), (2,5) and (5,2) which are obtained by coupling the 1^*st *^column of *TX *and the 2^*nd *^column of *TY*.

Since (3, 3) < (4, 6), (4, 6) can be omitted by pruning operation 1. On the next level, the successors of (3, 3), (2, 5) and (5, 2) are (4, 6), (5, 4), (5, 7) and (6, 6). Since identical pair (6, 6) is a successor of (5, 4), (5, 7) is a successor of (4, 6) and they are on the same level, (6, 6) and (5, 7) can be pruned.

#### Pruning Operation 2

If on the same level, there are two identical character pairs (*i*_1_, *j*) and (*i*_2_, *j*) satisfying *i*_1 _<*i*_2_, then (*i*_2_, *j*) can be pruned without affecting the correctness of the algorithm in obtaining the LCS of *X *and *Y*.

##### Rationale

The reason we can prune (*i*_2_, *j*) is as follows. Let the successors of (*i*_1_, *j*) be (*l*_2_, *j*_2_)≺(*l*_3_, *j*_3_)≺...≺(*l*_*r*_, *j*_*r*_), then the length of common subsequence of "*x*_*i*1_*x*_*i*1+1 _...*x*_*n*_" and "*y*_*j*_*y*_*j*+1 _...*y*_*m*_" is just *r*. Let the successors of (*i*_2_, *j*) are (*k*_2_, *j*_2_')≺(*k*_3_, *j*_3_')≺...≺(*k*_*q*_, *j*_*q*_'). Because i1 < i2, if an LCS contains a subsequence following (i2, j), this exact subsequence(note that all the x index is larger than i2) can be added at the end of (i1, j). Since (i1, j) and (i2, j) are on the same level, there must exist an LCS containing (i1, j). In other words, for any LCS containing identical character pair (i2, j), there is at least a corresponding LCS containing identical character pair (i1, j). Thus (*i*_2_, *j*) can be pruned without affecting the algorithm to get the LCS of *X *and *Y*.

By extending pruning operation 2, we can get the following pruning operation.

#### Pruning Operation 3

If there are identical character pairs (i_1_, j), (i_2_, j), ...,(i_*r*_, j) and *i*_1 _<*i*_2_<...<*i*_*r*_, then we can prune(*i*_2_, *j*),...,(*i*_*r*_, *j*).

### Framework of FAST_LCS and complexity analysis

Based on the operations of generating the successors of the identical character pairs using successor tables and the pruning operations, we present a fast parallel LCS algorithm named FAST_LCS. The algorithm consists of two phases: searching for all the identical character pairs and tracing back to get the LCS. The first phase begins with the initial identical character pairs; then continuously searches for successors using the successor tables. In this phase, the pruning technology is implemented to discard those search branches that obviously can not obtain the optimum solution so as to reduce the search space and speed up the process of searching.

The framework of the FAST_LCS algorithm is as follows, where the phase of searching for all the identical character pairs consists of steps 1, 2, 3 and the phase of tracing back is in step 4.

Step 1. Build tables *TX *and*TY*;

Step 2. Find all the initial identical character pairs:(*TX*(*k*, 0),*TY*(*k*, 0)), where *k *= 1,2,3,4, and add the records of the initial identical pairs (*k*, *TX*(*k*, 0), *TY*(*k*, 0), 1, *Φ*, *active*), *k *= 1,2,3,4 to the table *pairs*.

Step 3. Repeat the following until there is no record in *active *state in table *pairs*.

Step 3.1 For all *active *identical pairs (*k*, *i*, *j*, *level*, *pred*, *active*) in *pairs *parallel-do

Step 3.1.1 Produce all the direct successors of (*k*, *i*, *j*, *level*, *pred*, *active*).

Step 3.1.2 For each identical character pair (*g*, *h*) in the direct successors set of (*k*, *i*, *j*, *level*, *pred*, *active*), a new record (*k'*, *g*, *h*, *level*+1, *k*, *active*) is generated and inserted into the table *pairs*.

Step 3.1.3 Change the state of (*k*, *i*, *j*, *level*, *pred*, *active*) into *inactive*.

Step 3.2 Use the pruning operations on all the successors produced on this level to remove all the redundant identical pairs from table *pairs*.

Step 4. Compute *r *= the maximum level in the table *pairs*.

For all the identical pairs (*k*, *i*, *j*, *r*,*l*,*inactive*) in *pairs *parallel-do

Step 4.1. *pred *= *l*; *LCS*(*r*) = *x*_*i*_.

Step 4.2 While *pred *≠ *Φ *do

Step 4.2.1 get the *pred*-th record (*pred*, *g*, *h*, *r*',*l*', *inactive*) from table *pairs*.

Step 4.2.2 *pred *= *l*'; *LCS*(*r*') = *x*_*g*_.

In the algorithm, a table called *pairs *is used to store the identical character pairs obtained in the algorithm. In the table *pairs*, each record takes the form of (*k*, *i*, *j*, *level*, *pred*, *state*) where the data denote the index of the record, the identical character pair (*i*, *j*), its level, the index of its direct predecessor, and its current state, respectively. Each record in *pairs *has two states. For the identical pairs whose successors have not been searched, it is in the *active *state, otherwise it is in the i*nactive *state. In every step of search process, the algorithm searches for the successors of all the identical pairs in *active *state in parallel, and repeat this search process until there is no more identical pairs in *active *state in the table. The phase of tracing back starts from the identical pairs with the maximum level in the table, and traces back according to the *pred *of each identical pair. This tracing back process ends when it reaches an initial identical pair, and the trail indicates the LCS. If there are more than one identical pairs with the maximum level in the table, the tracing back procedure for those identical pairs can be carried out in parallel and several LCS can be obtained concurrently.

The LCS of *X*, *Y *is stored in the array *LCS*. In our algorithm, every identical pair must have the operation of producing successors at least once. Because of the pruning technology, this operation will only be run exactly once on each identical character pair. Therefore, assuming that the number of the identical character pairs of *X*, *Y *is *L*, the time complexity for a sequential execution of the algorithm FAST_LCS (*X*, *Y*) is *O*(*L*). Since the table *pairs *has to store all the identical character pairs, it requires *O*(*L*) memory space. Considering that the space cost of *TX*, *TY *are 4*(*n*+1) and 4*(*m*+1), the storage complexity of our algorithm is max{4*(*n*+1)+4*(*m*+1), *L*}. In parallel implementation of the algorithm, since the process for each identical pair can be assigned to one processor, all the operations on the identical pairs can be carried out in parallel. Thus, the processing of each level requires *O*(1) time, and the number of time steps required for a parallel execution of FAST_LCS is equal to the maximum level of the identical pairs. By Theorem 1, we know that the length of the LCS of *X*, *Y*, |LCS (*X*, *Y*)|, is equal to the maximum level of the identical pairs. Therefore, the time complexity of parallel FAST_LCS is *O*(|LCS(*X*,*Y*)|).

### Finding the LCS of multiple sequences using FAST_LCS

Our algorithm FAST_LCS can be easily extended to the LCS problem of multiple sequences. Suppose there are *n *sequences *X*_1_, *X*_2_, ..., *X*_*n*_, where *X *= (*x*_*i*1_, *x*_*i*2_, ..., *x*_*i*,*ni*_), *n*_*i *_is the length of *X*_*i*_, *x*_*ij *_∈ {*A*, *C*, *G*, *T*} where j = 1, 2, ..., *n*_*i*_. To find their LCS, similar to the case of two sequences, the algorithm for multiple sequences first builds the successor tables for all the sequences. Denote the successor tables of *X*_1_, *X*_2_, ..., *X*_*n *_as *TX*_1_, *TX*_2_, ..., *TX*_*n*_, respectively, where *TX*_*s *_is a two-dimensional array of size 4*(*n*_*s *_+ 1 [-del-n_*i*_+1]) for the sequence *X*_s _= (*x*_*s*1_, *x*_*s*2_, ..., *x*_*s*,*ns*_), *s *= 1,2,...*n*, and successor table *TX*_*s *_of identical characters is defined as:



where *SX*_*s *_(*i*, *j*) = {*k*|*x*_*sk *_= CH(*i*), *k*>*j*} where i = 1, 2, 3, 4.

Similar to identical character pairs in the case of two sequences, we define the concept of ***identical character tuple ***for LCS of multiple sequences. For the sequences *X*_1_, *X*_2_, ..., *X*_*n*_, if ,we record them in an identical character tuple of the sequences *X*_1_, *X*_2_, ...,*X*_*n *_and denote it as (*i*_1_, *i*_2_, ..., *i*_*n*_). The set of all the identical character tuples of *X*_1_, *X*_2_, ...,*X*_*n *_is denoted as *S*(*X*_1_, *X*_2_, ..., *X*_*n*_).

Let (*i*_1_, *i*_2_, ..., *i*_*n*_) and (*j*_1_, *j*_2_, ...,*j*_*n*_) be two identical character tuples of *X*_1_, *X*_2_, ..., *X*_*n*_. If *i*_*k *_<*j*_*k*_, for *k *= 1,2,...*n*, we call (*i*_1_, *i*_2_, ..., *i*_*n*_) a predecessor of (*j*_1_, *j*_2_, ..., *j*_*n*_), or (*j*_1_, *j*_2_, ..., *j*_*n*_) a successor of (*i*_1_, *i*_2_, ..., *i*_*n*_), and denote them as (*i*_1_, *i*_2_, ..., *i*_*n*_) < (*j*_1_, *j*_2_, ..., *j*_*n*_).

Let *P*(*i*_1_, *i*_2_, ..., *i*_*n*_) = {(*j*_1_, *j*_2_, ..., *j*_*n*_) | (*i*_1_, *i*_2_, ..., *i*_*n*_) < (*j*_1_, *j*_2_, ..., *j*_*n*_), (*j*_1_, *j*_2_, ..., *j*_*n*_)∈*S*(*X*_1_, *X*_2_, ..., *X*_*n*_)} be the set of all the successors of identical tuple (*i*_1_, *i*_2_, ..., *i*_*n*_), if (*k*_1_, *k*_2_, ...,*k*_*n*_)∑*P *(*i*_1_, *i*_2_, ..., *i*_*n*_) and there is no (*k*_1_', *k*_2_', ..., *k*_*n*_')∈*P*(*i*_1_, *i*_2_,..., *i*_*n*_) satisfying the condition: (*k*_1_', *k*_2_', ..., *k*_*n*_') < (*k*_1_, *k*_2_, ..., *k*_*n*_) and , we call (*k*_1_, *k*_2_, ..., *k*_*n*_) the direct successor of (*i*_1_, *i*_2_, ..., *i*_*n*_), and denoted it as (*i*_1_, *i*_2_, ..., *i*_*n*_)≺(*k*_1_, *k*_2_, ..., *k*_*n*_).

If an identical tuple (*i*_1_, *i*_2_, ..., *i*_*n*_)∈*S*(*X*_1_, *X*_2_, ..., *X*_*n*_) and there is no (*k*_1_, *k*_2_, ..., *k*_*n*_)∈*S*(*X*_1_, *X*_2_, ..., *X*_*n*_) satisfying (*k*_1_, *k*_2_, ..., *k*_*n*_)≺(*i*_1_, *i*_2_, ..., *i*_*n*_), we call (*i*_1_, *i*_2_, ..., *i*_*n*_) an initial identical tuple.

For an identical tuple (*i*_1_, *i*_2_, ..., *i*_*n*_)∈*S*(*X*_1_, *X*_2_, ..., *X*_*n*_), its level is defined as follows:



Similar to the case of two sequences LCS, the following theorems can be easily deduced.

**Theorem 3. **Denote the length of the LCS of *X*_1_, *X*_2_,..., *X*_*n *_as |LCS(*X*_1_, *X*_2_,...,*X*_*n*_)|, then

|LCS(*X*_1_,*X*_2_,...,*X*_*n*_)| = max {*level*(*i*_1_,*i*_2_,...*i*_*n*_)|(*i*_1_,*i*_2_,...*i*_*n*_)∈*S*(*X*_1_,*X*_2_,...,*X*_*n*_)}.

Proof of Theorem 3 is similar to that of Theorem 1.

In our parallel algorithm for LCS of multiple sequences, all direct successors of all the initial identical character tuples are produced in parallel using the successor tables. Then, the direct successors of all those successors produced in the first step are generated in parallel. Repeat these operations of generating the direct successors until no more successors could be produced. For an identical character tuple (*i*_1_, *i*_2_, ..., *i*_*n*_) ∈ *S *(*X*_1_, *X*_2_,..., *X*_*n*_), this operation is as follows:

(*i*_1_, *i*_2_,...,*i*_*n*_)→{(*TX*_1_(*k*, *i*_1_), *TX*_2_(*k*, *i*_2_),...,*TX*_*n*_(*k*, *i*_*n*_))| *k *= 1,2,3,4, *TX*_*j*_(*k*, *i*_*j*_) ≠ '-', *j *= 1,2,...,*n*}     (6)

From (6) we can see that this operation is to assemble the elements of the *i*_*j*_-th column of *TX*_*j*_, for *j *= 1,2,...,*n *to get the new tuples.

**Example 2. **Let *n *= 3, and *X*_1 _= "T G C A T A", *X*_2 _= "A T C T G A T", and *X*_3 _= "C T G A T T C". Their successor tables *TX*_1_, *TX*_2 _and *TX*_3 _are shown in Table 2.

By assembling the 0^th ^columns of the successor tables, we can get the initial identical triples (4,1,4), (3,3,1), (2,5,3) and (1,2,2).

The direct successors of the identical character triple (1,2,2) can be obtained by grouping the elements of the 1^st ^column of *TX*_1 _and the 2^nd ^columns of *TX*_2 _and *TX*_3_, which are (4,6,4),(3,3,7) (2,5,3) and (5,4,5).

**Theorem 4. **For an identical character tuple (*i*_1_, *i*_2_, ..., *i*_*n*_), the method in (6) can produce all its successors.

Proof of Theorem 4 is similar to that of Theorem 2.

From Theorem 4, we know that all the successors of the identical tuples on each level can be generated by the operation of producing successors. Starting from the initial identical tuples, all the identical tuples can be produced. In such process of generating the successors, pruning techniques can be implemented to remove the identical tuples which can not generate the longest common subsequence so as to reduce the search space. All the pruning operations for two-sequence LCS can be easily extended to the case of multiple sequences.

For instance, in Example 2, among the successors of triple (1,2,2), we have (2,5,3)≺(4,6,4), since they are on the same level, we can prune (4,6,4). For another instance in Example 2, among the initial identical triples (4,1,4), (3,3,1), (2,5,3) and (1,2,2), since they are in the same level and (1,2,2)≺(2,5,3) we can prune (2,5,3).

Assume that the number of the identical character tuples of the sequences *X*_1_, *X*_2_, ..., *X*_*n *_is *L*. In our algorithm, since every identical tuple must have the operation of producing successors exactly once, the time complexity for sequentially executing of the algorithm on the sequences *X*_1_, *X*_2_, ..., *X*_*n *_is *O*(*L*). The algorithm uses a table *tuples *to store all the identical character tuples, it requires *O*(*L*) memory space. Considering that the memory space cost of *TX*_*j*_, for *j *= 1,2,...,*n*, is 4, the storage complexity of our algorithm is max{4 , *L*}. In a parallel implementation, since the computation for each identical tuple can be assigned on one processor, all the process on the identical tuples can be carried out in parallel. Therefore, the processing of each level requires *O*(1) time, and the time required for the parallel computation is equal to the maximum level of the identical tuples which is equal to the length of the longest common subsequence of *X*_1_, *X*_2_, ..., *X*_*n*_. Therefore the time complexity of the parallel execution of FAST_LCS on multiple sequences is *O*(|LCS(*X*_1_, *X*_2_, ..., *X*_*n*_)|).

It should be pointed out that for most of the algorithms for multiple-sequence LCS, their time complexity strongly depends on the number of the sequences. For instance, if we use the Smith-Waterman algorithm to find the LCS of multiple sequences, the time complexity is , where *n *is the number of sequences, which is not practicable when *n *is large. The time complexity of our algorithm is *O*(*L*) for sequential computation and *O *(|LCS(*X*_1_, *X*_2_, ..., *X*_*n*_)|) for parallel implementation, where the time complexity of FAST_LCS is ***independent of the number of sequences n***. This means that our algorithm is much more efficient for finding the LCS of a large number of sequences.

## Results

### The results of sequential computation on two sequences

We test our algorithm FAST_LCS on the rice gene sequences of the *tigr *[29] database and compare the performance of FAST_LCS with that of Smith-Waterman algorithm [30] and FASTA algorithm [31,32] which are currently the most widely used LCS algorithms. Since both our algorithm and Smith-Waterman's can obtain exactly correct solution, we compare the computation speed of our algorithm FAST_LCS with that of Smith-Waterman algorithm. Also, we compare the precision of our algorithm with that of FASTA using the same computation time.

Table 3 compares the computation speed of FAST_LCS with that of Smith-Waterman algorithm on groups of gene sequence pairs with different lengths. Since a test on one pair of sequences takes very short time, it is hard to compare the speed of the algorithms using a single pair of sequence. Therefore we test the algorithms on groups of sequence pairs with similar lengths. We test five groups of sequence pairs each of which consisting of 100 pairs of sequences. The total time for each group by the two algorithms are listed in Table 3.

**Figure 1:**
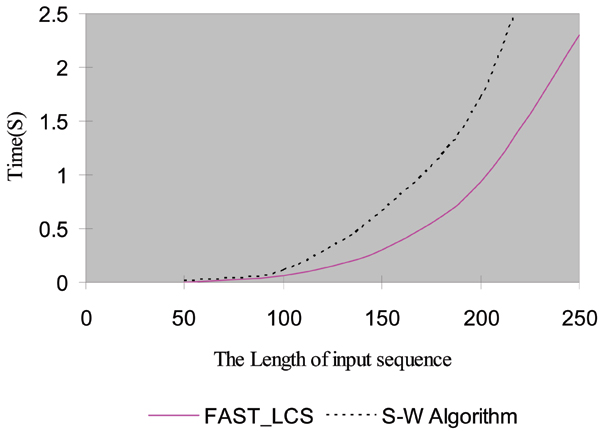
Comparison of the computation time of FAST_LCS with that of Smith – Waterman algorithm.

Fig. 1 shows the comparison of the computation time of our algorithm with that of Smith-Waterman algorithm. From the table and the figure, we can see that our algorithm is obviously faster than Smith-Waterman algorithm for sequence sets of all different lengths. The difference of the computation time between the two algorithms grows exponentially when the length of sequences becomes greater than 150. This means that our algorithm is much faster and more efficient than Smith-Waterman's for finding the LCS of long sequences.

**Figure 2:**
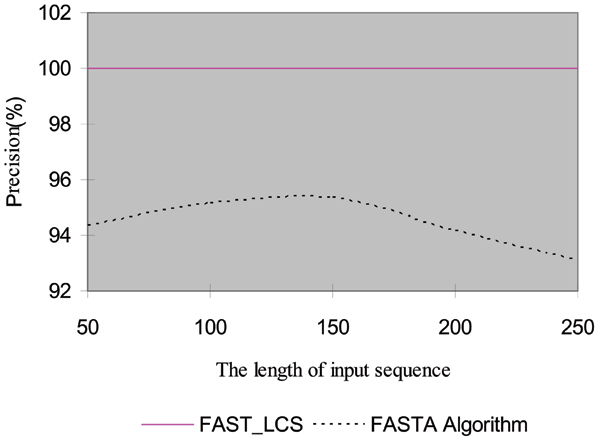
Comparison of the precision of FAST_LCS with that of FASTA using the same computation time.

We also compare the precision of our algorithm with that of FASTA on the premise of the same computing time. Here precision is defined as:



Fig. 2 shows the comparison of the precision of the results by our algorithm with that by FASTA using the same computation time. From Fig. 2, we can see that our algorithm can obtain the correct result no matter how long the sequence is, while the precision of FASTA declines when the length of the sequences is increased. Therefore the precision of our algorithm is much higher than that of FASTA.

### The results of sequential computing on multiple sequences

**Figure 3:**
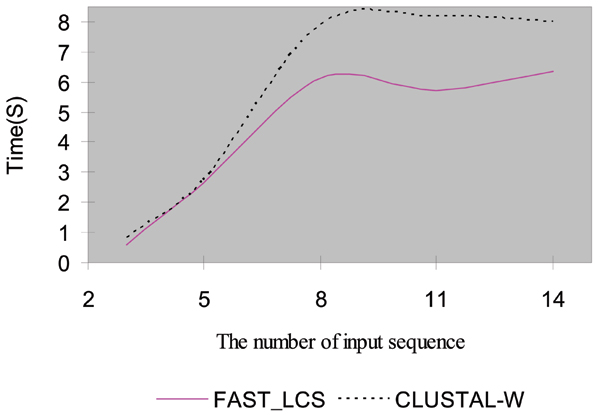
Comparison of the computation time of FAST_LCS with that of Clustal-W on sequence sets of different numbers of sequences.

**Figure 4:**
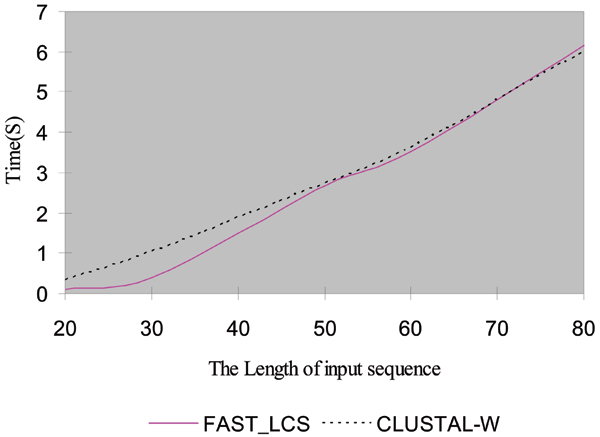
Comparison of the computation time of FAST_LCS with that of Clustal-W on sequence sets of different lengths.

We test our algorithm FAST_LCS on the multiple sequences and compare with the Clustal-W[27] algorithm which is a popular algorithm for multiple-sequence LCS. Fig. 3 and Fig. 4 show the comparison of the computation time of our algorithm FAST_LCS with that of Clustal-W algorithm. Table 4 lists the computation time of the two algorithms on 5 sets of different numbers of sequences with length of 50. Comparison of the computation time of the two algorithms on sequences sets of different numbers of sequences is shown in Fig. 3.

From Fig. 3 and Table 4, we can see that FAST_LCS is faster than Clustal-W for sets with different numbers of sequences. When the number of sequences is larger than five, the speed up is significant.

Table 5 lists the computation time of the two algorithms on five sequences sets with different lengths. Comparison of the computation time of the two algorithms on sequence sets of different lengths is shown in Fig. 4. From Table 5 and Fig. 4, we can see that FAST_LCS is faster than Clustal-W for sequence sets with different lengths.

**Figure 5:**
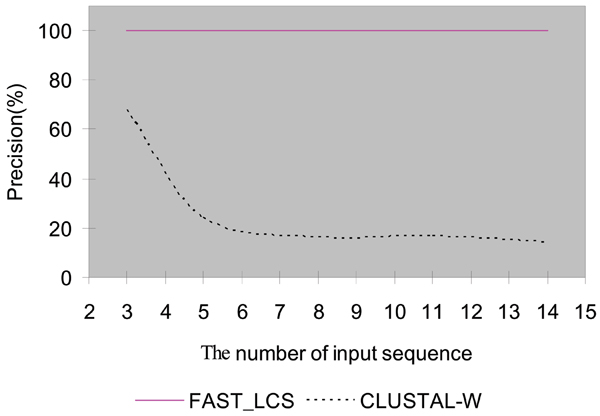
Comparison of the precision of FAST_LCS with that of Clustal-W on sequence sets with different numbers of sequences.

**Figure 6:**
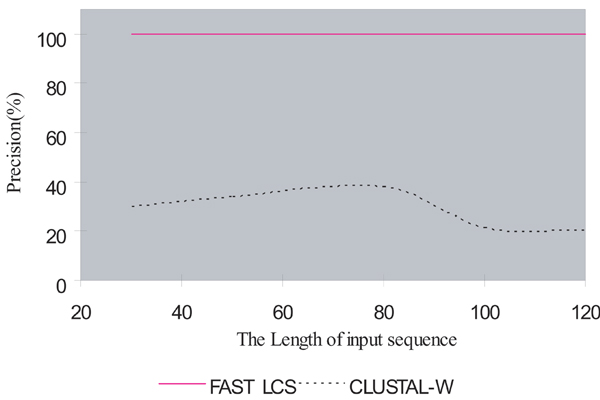
Comparison of the precision of FAST_LCS with that of Clustal-W on sequence sets of different lengths.

We also compare the precision of our FAST_LCS algorithm with that of Clustal-W algorithm. Fig. 5 shows the comparison of precision of FAST_LCS with that of Clustal-W on the sets with different numbers of sequences, and Fig. 6 shows the comparison of precision of the two algorithms on the sets of sequences with different lengths. From these two figures, we can see that no matter how the length and the number of sequences are increased, our algorithm can obtain the exactly correct results. The precision of Clustal-W declines when the number or the length of the sequences is increased. Therefore the precision of our algorithm is much higher than Clustal-W.

### The results of parallel computing

**Figure 7:**
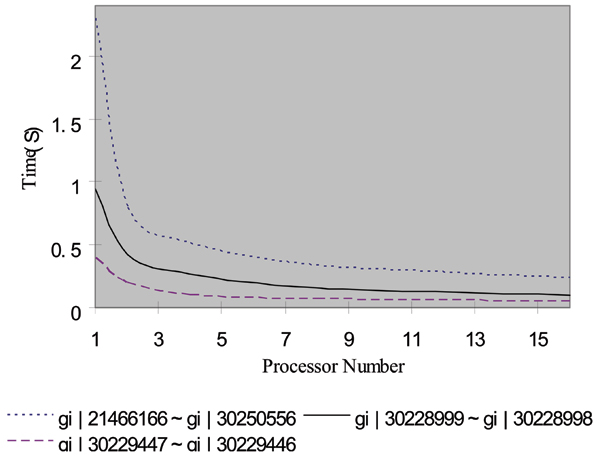
Parallel computational time of FAST_LCS using different processor numbers.

We also test our algorithm on the rice gene sequence from the *tigr *database [29] on the Shenteng 1800 supercomputer using MPI (C bounding). In the parallel implementation of FAST_LCS, the identical character pairs in the *active *state are assigned and processed in different processors. The experimental results by using different numbers of processors are shown in Fig. 7. Three pairs of gene sequences are tested. The names, lengths, and computation times are listed in Table 6. From Fig. 7 and Table 6 we can see that the computation will become faster as the number of processors increases. Due to the communication overhead between processors, the speedup of our algorithm is slower than linear, which conforms to the Amdahl's Law.

## Figures and Tables

**Table 1:** Their successor tables *TX *and *TY *in Example 1.

*TX*:		
*i*	CH(*i*)	0 1 2 3 4 5 6
1	**A**	4 4 4 4 6 6 -
2	**C**	3 3 3 - - - -
3	**G**	2 2 - - - - -
4	**T**	1 5 5 5 5 - -

*TY:*		

*i*	CH(*i*)	0 1 2 3 4 5 6 7
1	**A**	1 6 6 6 6 6 - -
2	**C**	3 3 3 - - - - -
3	**G**	5 5 5 5 5 - - -
4	**T**	2 2 4 4 7 7 7 -

**Table 2:** Their successor tables *TX*_1_, *TX*_2 _and *TX*_3 _in Example 2

*TX*_1_:		
*i*	CH(*i*)	0 1 2 3 4 5 6
1	**A**	4 4 4 4 6 6 -
2	**C**	3 3 3 - - - -
3	**G**	2 2 - - - - -
4	**T**	1 5 5 5 5 - -

*TX*_2_:		

*i*	CH(*i*)	0 1 2 3 4 5 6 7
1	**A**	1 6 6 6 6 6 - -
2	**C**	3 3 3 - - - - -
3	**G**	5 5 5 5 5 - - -
4	**T**	2 2 4 4 7 7 7 -

*TX*_3_:		

*i*	CH(*i*)	0 1 2 3 4 5 6 7
1	**A**	4 4 4 4 - - - -
2	**C**	1 7 7 7 7 7 7 -
3	**G**	3 3 3 - - - - -
4	**T**	2 2 5 5 5 6 - -

**Table 3:** Comparison of computation speed of FAST_LCS with that of Smith-Waterman algorithm

			Time of FAST_LCS (s)		Time of S-W algorithm(s)	
			
Name of Sequences	Length *l*	Number of pairs	Total time	Average time	Total time	Average time
gi|21466196 ~	0≤*l*≤50	100	0.49	0.0049	1.09	0.0109
gi|21466195 ~						
...						
gi|21466168 ~						
gi|21466167 ~						
gi|21466166 ~						
gi|30250556 ~						
gi|30230255 ~						
gi|30230254 ~						
gi|30229613 ~						
gi|30229612 ~						
...						
gi|30229449 ~						
gi|30229448 ~						

gi|30229047 ~	50≤ *l*≤ 100	100	5.88	0.0588	11.55	0.1155
gi|30229046 ~						
...						
gi|30229001 ~						
gi|30229000 ~						
gi|30228999 ~						
gi|30228998 ~						
...						
gi|30228849 ~						
gi|30228848 ~						

gi|30229447 ~	10≤ *l*≤ 150	100	29.41	0.2941	65.95	0.6595
gi|30229446 ~						
...						
gi|30229249 ~						
gi|30229248 ~						

gi|30228846 ~	15≤ *l*≤ 200	100	94.11	0.9411	172.213	1.7213
gi|30228845 ~						
...						
gi|30228648 ~						
gi|30228647 ~						

gi|30229247 ~	20≤ *l*≤ 250	100	230.51	2.3051	425.16	4.2516
gi|30229246 ~						
...						
gi|30229049 ~						
gi|30229048 ~						

**Table 4:** Comparison of computation time of FAST_LCS and that of Clustal-W on sequences sets of different numbers of sequences

Sequence name	Sequence Number	Time of FAST_LCS (s)	Time of Clustal-W (s)
gi|21466194	3	0.609	0.804
...			
gi|21466196			

gi|21466192	5	2.656	2.732
...			
gi|21466196			

gi|21466189	8	6.14	7.91
...			
gi|21466196			

gi|21466186	11	5.71	8.20
...			
gi|21466196			

gi|21466183	14	6.34	8.49
...			
gi|21466196			

**Table 5:** Comparison of computation time of FAST_LCS with that of Clustal-W on sequences sets of different lengths

	The length of input sequences				
	
Algorithm	20	30	50	60	80
Time of FAST_LCS (S)	0.109	0.391	2.656	3.516	6.166
Time of Clustal-W (S)	0.312	1.053	2.732	3.612	5.992

**Table 6:** Computational time of parallel FAST_LCS using different numbers of processors

		Computational time using different numbers of processors (s)				
		
Sequence name	Length	1	2	4	8	16
gi|21466166 ~	250	2.3051	0.7955	0.51085	0.3300	0.2313
gi|30250556						

gi|30228999 ~	200	0.9411	0.4247	0.2669	0.15421	0.09379
gi|30228998						

gi|30229447 ~	150	0.3941	0.2015	0.10271	0.06975	0.04702
gi|30229446						
